# How flower development genes were identified using forward genetic screens in *Arabidopsis thaliana*

**DOI:** 10.1093/genetics/iyad102

**Published:** 2023-06-09

**Authors:** David R Smyth

**Affiliations:** School of Biological Sciences, Monash University, Melbourne, VIC 3800, Australia

**Keywords:** Arabidopsis, flower development, mutant screen, homeotic mutants, ABC model, flower meristem identity, inflorescence development

## Abstract

In the later part of the 1980s, the time was ripe for identifying genes controlling flower development. In that pregenomic era, the easiest way to do this was to induce random mutations in seeds by chemical mutagens (or irradiation) and to screen thousands of plants for those with phenotypes specifically defective in floral morphogenesis. Here, we discuss the results of premolecular screens for flower development mutants in *Arabidopsis thaliana*, carried out at Caltech and Monash University, emphasizing the usefulness of saturation mutagenesis, multiple alleles to identify full loss-of-function, conclusions based on multiple mutant analyses, and from screens for enhancer and suppressor modifiers of original mutant phenotypes. One outcome was a series of mutants that led to the ABC floral organ identity model (*AP1*, *AP2*, *AP3*, *PI*, and *AG*). In addition, genes controlling flower meristem identity (*AP1*, *CAL*, and *LFY*), floral meristem size (*CLV1* and *CLV3*), development of individual floral organ types (*CRC*, *SPT*, and *PTL*), and inflorescence meristem properties (*TFL1*, *PIN1*, and *PID*) were defined. These occurrences formed targets for cloning that eventually helped lead to an understanding of transcriptional control of the identity of floral organs and flower meristems, signaling within meristems, and the role of auxin in initiating floral organogenesis. These findings in Arabidopsis are now being applied to investigate how orthologous and paralogous genes act in other flowering plants, allowing us to wander in the fertile fields of evo-devo.

## Teaser

Thirty-five years ago, the field set out to understand how genes controlled flower morphogenesis, relying on chemical mutagenesis-based forward genetic screens in Arabidopsis since molecular tools were not yet available. Here, Smyth reviews these screens, which led to the ABC model of floral organ identity; the uncovering of later sepal, petal and carpel developmental control; understanding how inflorescence and floral meristem identity are conferred; and identifying new signaling pathways. Now, molecular understanding of how these genes function is extending these early findings across Angiosperms.

## Introduction

Mendel (1866) established the transmission behavior of genes (determinants of traits) firstly as alternative forms of one gene and secondly in combinations of more than one gene. He succeeded because he chose to follow traits with alternative forms (variants) rather than traits that show continuous variation. In addition, he started out with true breeding lines that behaved predictably, generating only one form of a trait unless intercrossed with other lines.

Classical genetics relied at first on serendipity to provide variants to interbreed and follow down the generations. In the 1920s, it was discovered that variants (mutants) could be induced by treating organisms with X-irradiation (Muller 1927) or later by specific chemicals (Auerbach *et al*. 1947). Even though the recovery of variants was much increased over spontaneous levels, the production of specific variants was unpredictable and varied stochastically. In ground-breaking studies on the nature of the gene, Seymour Benzer in the 1950s focused on plaque morphology in bacteriophage T4 following chemical mutagenesis. He obtained hundreds of variants within 2 individual genes and showed that each gene (he called them cistrons) was mutable at many internal sites, either as point mutants or as deletions of adjoining sites (Benzer 1959). Later analysis of extensive mutation collections in bacteriophage T4 revealed that the structure of the mature phage was controlled by the sequential action of genes in branched linear pathways (Wood 1980), an early use of saturation mutagenesis to identify all genes involved in a specific morphogenetic process.

In *Drosophila melanogaster*, similar approaches were used to reveal how genes controlled morphogenesis. A successful example was that of Ed Lewis, who in the 1950s and 1960s, collected mutants that disrupted segmentation of the adult body. This led him to uncover several adjacent genes in the *Bithorax complex* that determined the identity of thoracic and abdominal segments. Their role was inferred because mutants resulted in changes in body segment identity, known as homeotic mutants (Lewis 1978). Subsequent large-scale mutation hunts in the 1970s focused on changes in embryo and larval segmentation (loss or identity changes) (Nüsslein-Volhardt and Wieschaus 1980; Irion and Nüsslein-Volhard 2022) and led to an understanding of how the sequential activity of the wild-type genes controlled each step. Similar screens in the nematode *Caenorhabditis elegans* had shown the way (Brenner 1974).

Meanwhile in plants, morphogenesis had been meticulously described at the macroscopic and cellular levels. But understanding the genetic control of these processes relied on rare sporadic mutants. Significant collections of mutants had been obtained especially in crop plants subject to intimate and long-term observation such as maize (Neuffer *et al*. 1968; Scandalios 1982; Sheridan 1988) and tomato (Stevens and Rick 1986). Large-scale collections in floricultural species including snapdragon (Stubbe 1966) and petunia (Cornu and Maizonnier 1983) were also curated. But the following were needed to help uncover the mechanism of the action of genes controlling morphogenesis: (1) allelic series indicating the consequences of full loss-of-function in recurring strongly affected alleles, (2) saturation mutagenesis to reveal most or all genes involved in specific developmental pathways, (3) multiple mutant combinations to deduce gene interactions (Table 1), and (4) second-site screens for enhancers and suppressors of the original mutant phenotype.

**Table 1.** How the respective roles of developmental genes X and Y can be deduced from their double loss-of-function mutant phenotype.

Class of interaction		Double mutant phenotype	Deduction
Additive	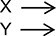	Double mutant combines phenotypes of single mutants	X and Y have different roles
Overlapping	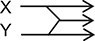	Double mutant is more disrupted than the additive phenotype	X and Y share part function
Epistatic	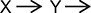	Double and X mutant the same, Y mutant different	X acts upstream of Y
Shared	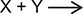	Double mutant the same as both X and Y single mutants	Both X and Y are required
Redundant	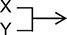	Double mutant is disrupted, X and Y single mutants wild type	Either X or Y is required

Examples, especially involving organ identity (ABCE) mutants, include: additive: *ap3* and *ag*; *pi* and *ag*; overlapping: *ap1* and *cal*; *spt* and *crc*; epistatic: *ag* and *crc*; *ag* and *spt*; shared: *pi* and *ap3; clv1/2/3*; redundant: *sep1/2/3/4*.

A plant model species with facile genetics analogous to *D. melanogaster* was needed. In the mid 1980s, the wall cress *Arabidopsis thaliana* (family Brassicaceae) was chosen by many with the expectation that genes could be identified and cloned relatively easily (Leutwiler *et al*. 1984; Meyerowitz and Pruitt 1985; Estelle and Somerville 1986; Meyerowitz 1987). Morphological mutants were induced in early studies, although their genetic analysis was not followed up (Reinholz 1946; Röbbelen 1962; McKelvie 1962). Various groups then set out to focus on the development of embryos (Mayer *et al*. 1991), roots (Benfey *et al*. 1993), trichomes (Hülskamp *et al*. 1994), and flowers (Komaki *et al*. 1988) by collecting morphologically defective mutants specifically disrupted in each process using forward genetic screens. At this time, the molecular nature of developmental genes in plants was unknown, and it was anticipated that assemblies of mutants targeting specific processes would provide targets for cloning, leading to an understanding of molecular mechanisms.

Several homeotic genetic disruptions of flower morphogenesis had already been obtained in *A. thaliana* by Koornneef (1983) at Wageningen, the Netherlands. In these, one type of floral organ was replaced by another usually found at a different site within the flower. Such homeotic mutants had already shown their worth in revealing organ identity genes in Drosophila (see above), and we set about studying their genetics and developmental biology in Arabidopsis. At first, we relied on Koornneef's mutants *agamous* (*ag*), *apetala1* (*ap1*), *ap2*, *ap3*, and *pistillata* (*pi*), each single alleles of their respective wild-type genes. None of these had disruptions elsewhere in the plant indicating flower-specific action (Bowman *et al*. 1989). But a fuller understanding of flower morphogenesis required further mutants of both these genes (Bowman *et al*. 1991) and others specifically affecting other processes in floral development. This paper reports the results of mutant screens of Arabidopsis carried out in 1988 at Caltech and several years subsequently at Monash University that focused on flower morphogenesis and how they helped build a foundation for our understanding of this process.

## Methods

Dry seed of Landsberg *erecta* background was treated with 40-mM aqueous ethyl methane sulphonate (EMS) with stirring at room temperature for 8 hours. At this dose, it is expected that each locus has about one chance in 1,000 of being mutated per treated cell (Koornneef *et al*. 1982). Seeds were washed in distilled water overnight and blotted dry before planting 9 seeds per 65 mm square pot. The seeds were incubated at ∼25°C in a growth room under continuous Cool White fluorescent light. Once the M1 plants bore mature seeds generated by self-pollination in up to 10 siliques on the primary inflorescence (∼6–8 weeks), whole plants were collected individually into paper bags and dried. These steps were carried out at the California Institute of Technology by Leslie Leutwiler and Bob Pruitt. These growth conditions illustrate the advantages of Arabidopsis in the ability to grow many plants in a small space with rapid production of seeds.

In 1988, M2 seeds of 555 individual M1 plants [arising from ∼1,110 treated meristematic cells in the embryo, assuming the inflorescence of an adult plant is derived from two cells per seed (Koornneef *et al*. 1982)] were planted out. Plants were observed at weekly intervals for 6 weeks from germination until seed set. For most families, 24 or 25 seeds were planted, yielding 16.21 ± 0.012 SE flowering progeny per family on average (64%, range 2–24). This is consistent with many seeds carrying homozygous recessive mutations resulting in embryonic lethality. Overall 9,142 plants were screened at the flowering stage. Seeds of mutant M2 plants, or, if sterile, at least 5 of their phenotypically normal sibs, were collected. Individual mutants were given an isolation number corresponding to the M2 family number (prefixed “S”).

The effectiveness of the mutagenesis was shown by the occurrence of bleached cotyledons (either white or yellow) in M2 families (Li and Rédei 1969). Among 302 of the families screened for this trait, 45 (14.9%) had at least one bleached seedling (17 white families, 28 yellow). These were likely to have arisen from mutation events in the many nuclear genes required for photosynthesis.

Mutant lines of interest (all recessive) were successively back-crossed to Landsberg *erecta* to segregate out second-site mutations. Allelism tests between new mutants and mutants of known genes were then carried out by scoring the phenotypes of F1 progeny (if mutant, allelism was inferred).

This screen was extensive but unlikely to have approached saturation. We scanned M2 plants derived from about 1,110 M1 cells and estimated that the mutation rate per gene was around 1 in 1,000 cells (see above). Thus, by chance many genes may not have been mutated even once. For this reason, further mutagenesis screens of similar design were carried out from 1989 by John Alvarez at Monash University, Melbourne. Differences included using 25-mM EMS for 13 hours rather than 40 mM for 8 hours, and 1 of the 4 batches of treated seeds was of Columbia background rather than Landsberg *erecta*. Also, an additional batch of Landsberg *erecta* seeds was mutagenized with 25 krad of gamma irradiation from a ^60^Co source.

## Results

### Homeotic floral organ (ABCE) mutants: *ap1*, *ap2*, *ap3*, *pi*, *ag*, and *sep*

Our initial study of homeotic mutants was limited to one recessive mutant allele each of *AG*, *AP2*, *AP3*, and *PI* genes kindly provided by Maarten Koornneef (Koornneef 1983; Bowman *et al*. 1989). Based on single and double mutant phenotypes, these were sufficient to deduce that the wild-type genes act in combination to “allow cells to determine their place in the developing flower and thus to differentiate appropriately”, and that the 4 genes may act “in setting up or responding to concentric, overlapping fields within the flower primordium” (Bowman *et al*. 1989). On obtaining further alleles in the mutant hunt (Table 2), it was possible to deduce the full loss-of-function mutant phenotype (a strong phenotype shared by more than one allele) (Bowman *et al*. 1991). Interactions between homeotic mutants of all 4 genes, especially null mutants, were also studied by generating all gene combinations that were essential in developing the ABC model (Bowman *et al*. 1991, 2012).

**Table 2.** Flower morphogenetic mutants identified in EMS screens.

Gene	Mutant Allele No.*^a^*	Encoded Protein*^b^*	References
*AGAMOUS (AG)*	3, [4]	MADS TF (C)	Bowman *et al*. (1991), Sieburth *et al*. (1995)
*APETALA1 (AP1)*	[2–5, 7]	MADS TF (A)	Bowman *et al*. (1993)
*APETALA2 (AP2)*	2, 8, 9	AP2 TF (A)	Bowman *et al*. (1991)
*APETALA3 (AP3)*	None obtained	MADS TF (B)	Bowman *et al*. (1991)
*CAULIFLOWER (CAL)*	[1*^c^*]	MADS TF	Bowman *et al*. (1993)
*CLAVATA1 (CLV1)*	S292, S368	LRR-RLK	unpublished
*CLAVATA3 (CLV3)*	1, [2*^d^*]	CLE signaling peptide	Clark *et al*. (1995)
*CRABS CLAW (CRC)*	1, [2*^d^*]	YABBY TF	Alvarez and Smyth (1999, 2002), Bowman and Smyth (1999)
*LEAFY (LFY)*	3, 4 [5, 6, 10*^e^*, 11]	LEAFY TF	Weigel *et al*. (1992)
*LEUNIG (LUG)*	8, 9	TUP1 co-repressor	Liu and Meyerowitz (1995)
*PETAL LOSS (PTL)*	[1*^e^*, 3–5]	Trihelix TF	Griffith *et al*. (1999), Brewer *et al*. (2004)
*PETAL LOSS MODIFIER (PMD)*	[1*^f^*]	unknown	Griffith *et al*. (1999)
*PIN FORMED1 (PIN1)*	[3, 5]	Auxin efflux carrier	Bennett *et al*. (1995)
*PINOID (PID)*	1, [2, 3*^e^*, 4, 7]	S/T protein kinase	Bennett *et al*. (1995)
*PISTILLATA (PI)*	2, 3	MADS TF (B)	Bowman *et al*. (1991)
*SEPALLATA1-4 (SEP1-4)*	None obtained	MADS TF (E)	
*SPATULA (SPT)*	1, [2, 3]	bHLH TF	Alvarez and Smyth (1999, 2002), Heisler *et al.* (2001)
*SUPERMAN (SUP)*	[4*^e^*]	C2H2 ZnF TF	Bowman *et al.* (1992)
*TERMINAL FLOWER1 (TFL1)*	2–5, [6, 7, 8*^e^*]	CETS	Alvarez *et al*. (1992)
*UNUSUAL FLORAL ORGANS (UFO)*	[3–5]	F-box	Levin and Meyerowitz (1995)

Obtained at Caltech in 1988, or at Monash University in 1989–1992 (square brackets).

Determined subsequently. TF—transcription factor; A, B, C, E—floral organ identity class.

In Ws background.

Induced by gamma irradiation.

Induced in Col background, all others in L*er* background.

Present as a dominant modifier in L*er* background.

**Fig. 1.**
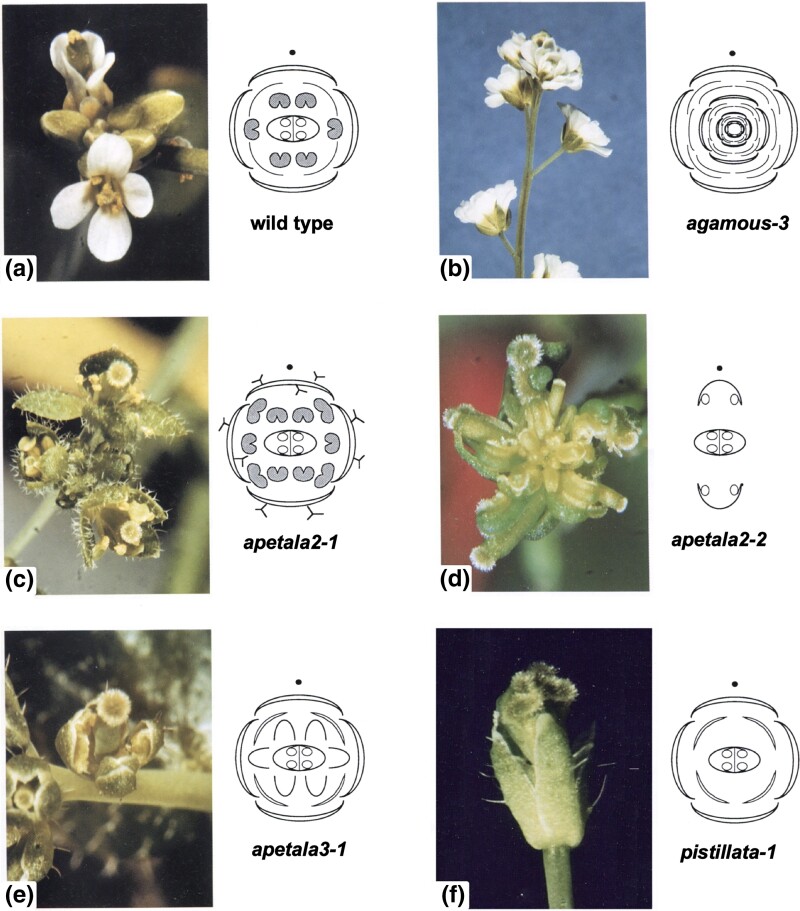
Inflorescences of wild-type and ABC mutants. a) Wild-type (Landsberg *erecta*). b) *ag-3* mutant (C function), closely similar to *ag-1* and thus likely to reflect full loss-of-function. c) *ap2-2* mutant (A function), an atypical allele with leaf-like sepals. d) *ap2-2* mutant with more severe disruptions same as those of several other alleles. e) *ap3-1* mutant (B function), with a weak phenotype. f) *pi-1* mutant (B function), with a strong phenotype. a–e) modified from Meyerowitz *et al*. (1989); f) modified from Bowman *et al*. (1989).

For instance, an additional allele of *AG*, *ag-3*, had a strong phenotype identical to *ag-1* indicating the full loss-of-function phenotype (Fig. 1b). A partial loss-of-function allele, *ag-4*, was later useful in separating out components of AG function in the third and fourth floral whorls (Sieburth *et al*. 1995). Also, later generation of second-site mutants in the *ag-4* background led to the discovery that microRNAs regulate the lifetime of mRNA transcripts of some of the interacting genes (Chen and Meyerowitz 1999).

For *AP2*, the original allele *ap2-1* was clearly atypical when further alleles were obtained by us [*ap2-2*, *8*, and *9*, Bowman *et al*. (1991)] (Fig. 1c and d), and others [*ap2-3* and *4*, Okada *et al*. (1991) and *ap2-5*, *6*, and *7*, Kunst *et al*. (1989)]. *ap2-2* and *ap2-8* shared the same strong abnormal phenotype pointing to the full loss-of-function phenotype.

For *PI*, 2 new mutant alleles *pi-2* and *pi-3* were less abnormal than *pi-1* (Fig. 1f) indicating that they, at least, had only partially lost PI function (Bowman *et al*. 1991). The weakest *pi* allele, *pi-3*, resembled *ap3-1* (Fig. 1e) suggesting these 2 genes had a shared function (Bowman *et al*. 1991) (Table 1). This was confirmed subsequently when 3 further *ap3* alleles were obtained by others that had the same strong phenotype as each other and *pi-1* (Jack *et al*. 1992), presumably resulting from full loss of AP3 or PI function.

The *AP1* gene was followed up later when an allelic series of 5 mutants was obtained (Bowman *et al*. 1993) (Table 2). The phenotype of one of these, *ap1-7*, matched the strong phenotype of the original allele, *ap1-1* (Koornneef 1983), likely indicating full loss-of-function. Going by mutant combinations, we proposed that one function of AP1 was to confer A function in combination with AP2 in defining the identity of the 2 outer floral whorls (Bowman *et al*. 1993).

The identity of all 4 floral organ types was shown subsequently by Marty Yanofsky's group to be controlled by 4 paralogous *SEPALLATA* (*SEP*) genes (Pélaz *et al*. 2000; Ditta *et al*. 2004), thus defining E function. These were identified using a reverse genetics approach. [Reverse genetics requires knowledge of the molecular nature of a gene (and its relatives if appropriate) before manipulating its function. Forward genetics involves modifying the function of a gene, usually by mutation, as a precursor to identifying its molecular nature]. In this case, 4 close relatives of AG from the MADS transcription factor family were each mutated by insertions and identified using a transgenic approach. Single mutants were phenotypically normal, and only quadruple mutants (*sep1 sep2 sep3 sep4*) had leaf-like organs in all floral whorls. Presumably, this redundancy (Table 1) accounts for why no mutants of *SEP* genes were identified in the forward genetics screens.

### Further mutants affected in organ identity: *sup*, and *lug*

The identity of fourth whorl organs as carpels is lost in *superman* (*sup*) mutants (Bowman *et al*. 1992), and termination of the floral meristem is delayed. This results in many additional stamens in place of carpels. One *sup* allele was obtained in our screen, *sup-4*, with a phenotype similar to the other 3 known at that stage (Table 2). It was proposed that SUP represses AP3 and PI (B) function in the fourth whorl, consistent with the localization of transcripts to the inside of the whorl 3/4 boundary (Prunet *et al*. 2017). Thus, *SUP* is not a primary organ identity gene, but one that blocks B function in a spatially defined region of the floral meristem. It is also involved along with AG in terminating the flower meristem after 2 carpel primordia have been generated. Later studies of weaker *sup* mutant alleles uncovered the general role of DNA methylation in epigenetic silencing of gene expression in plants (Jacobsen *et al*. 2000).

Other mutants were also obtained with less clear-cut effects on floral organ identity, and with additional defects elsewhere in the plant. Two were alleles of a gene named *LEUNIG* (*LUG*) (*lug-8* and *9*) after Michael Leunig, the creator of the cartoon character “Mr Curly” (Table 2). This reflected the curled apical extension of the carpels characteristic of such mutants [including *lug-10* (Fl89 of Komaki *et al*. 1988)]. Additional alleles were subsequently isolated as enhancers of identity defects in sepals and petals in the weak A function mutant *ap2-1* (Liu and Meyerowitz 1995). *lug* single mutants had similar but less severe identity changes to sepals and petals, leading the authors to propose that LUG constrained AG function somewhat in these whorls.

### Flower meristem identity mutants: *ap1*, *cal*, *lfy*, and *ufo*

AP1 is also involved earlier in defining the identity of the flower meristem itself. It does this partly in combination with a closely related MADS paralog *CAULIFLOWER* (*CAL*) (Bowman *et al*. 1993; Kempin *et al*. 1995) (Table 2). Double mutants of *ap1* and *cal* produce inflorescence primordia that generate further inflorescence primordia on their flanks rather than flower primordia, and so resemble dwarf “cauliflowers”. Mutants of the *cal* gene alone are phenotypically normal, so its loss-of-function is only revealed if AP1 function is also lost. That is, AP1 wild-type function fully overlaps with that of CAL, but not vice versa (Table 1). The *ap1 cal* double mutant proved useful in obtaining synchronized samples of developing flower buds from the earliest stages for transcriptional profiling. By incorporating an inducible *AP1* construct, the many newly arising meristems can be switched to develop as flower meristems rather than as inflorescence meristems (Wellmer *et al*. 2006).

Another key gene that defines flower meristem identity together with AP1 and CAL encodes the transcription factor LEAFY (LFY) (Weigel *et al*. 1992; Bowman *et al*. 1993). An allelic series of 6 *lfy* mutant alleles was obtained with a strengthening range of abnormal phenotypes [2 weak alleles (*lfy-5* and *10*), 2 intermediate (*lfy-3* and *4*), and 2 strong (*lfy-6* and *11*)] (Table 2). The phenotypes of the strong alleles were closely similar to *lfy-1* (Schultz and Haughn 1991), presumably revealing the consequences of full loss-of-function. Again, multiple mutant combinations of *lfy* with *ap1* and *cal* allowed their overlapping roles to be deduced (Weigel and Meyerowitz 1993).

A further gene with a role in defining floral meristem identity was *UNUSUAL FLORAL ORGANS* (*UFO*) (Wilkinson and Haughn 1995). Three mutants obtained here (*ufo-3*, *4*, and *5*) (Table 2) were closely similar in phenotype to each other and to 5 of 6 other independent mutant occurrences studied by Levin and Meyerowitz (1995). UFO is involved in conferring sepal and petal identity, boundary formation and determinacy of the flower meristem, sharing the first function at least with LFY (Levin and Meyerowitz 1995).

### Floral organogenesis mutants: *spt*, *crc*, and *ptl*

**Fig. 2.**
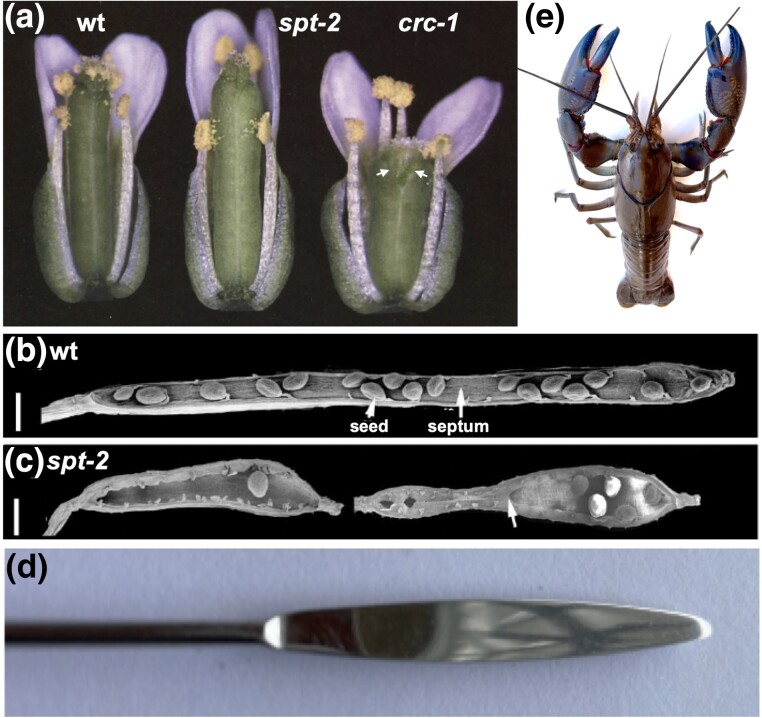
Mature flowers of wild type (wt), *spatula-2* and *crabs claw-1* mutants. a) The gynoecia of *spt-2* flowers are longer than wild type, with unfused carpels at the apex and no transmitting tract in the reduced septum. Those of *crc-1* mutants are shorter, wider, and the 2 unfused carpels are bent inward at the apex like a crab's claw (arrows). b) A wild-type silique with one valve removed showing seeds and septum. c) Two *spt-2* siliques showing reduced seed set and the septum absent (left) or much reduced (right). Bars represent 500 μm. d) A spatula after, which the gene was named. e) A YABBY, an Australian freshwater crayfish and a relative of the crab, after which the YABBY transcription factor family was named (Bowman and Smyth 1999). a–c) modified from Alvarez and Smyth (2002).

One category of mutant was identified as being defective in the development of one floral organ type. Gynoecium-only mutants were chosen as potentially acting downstream of the carpel identity gene *AG* (Alvarez and Smyth 1999, 2002). One of these had siliques wider in the medial plane, especially at the apex, and the gene was named *SPATULA* (*SPT*) after the laboratory and kitchen utensil (Table 2; Fig. 2a–d). Three alleles were obtained, with the stronger *spt-2* and *spt-3* alleles having carpels unfused at the apex and lacking a transmitting tract.

Another mutant class had shorter siliques with the 2 carpels unfused at the apex and bending inwards; hence, it was named *CRABS CLAW* (CRC) (Fig. 2a). Two alleles were identified, the stronger *crc-1* allele having more pronounced defects in the gynoecia that were shorter and wider than normal, and also lacked nectaries (Table 2). Additional alleles were identified later and revealed that *crc-1* was the strongest loss-of-function phenotype (Bowman and Smyth 1999). When *spt-2* and *crc-1* were combined, the gynoecia were abnormal in a nonadditive fashion, indicating that they have partially overlapping roles (Table 1). In addition, when combined as quadruple mutants with *ap2 ag* mutants (lacking A and C functions), the residual carpelloid properties of *ap2 ag* double mutants were lost, indicating that both SPT and CRC have a role in controlling aspects of carpel structure independently of AG (Alvarez and Smyth 1999).

A second category of floral organ mutants affected only sepals and petals, although not their identity. The most obvious phenotype was loss of petals, and hence, the gene was named *PETAL LOSS* (*PTL*) (Griffith *et al*. 1999) (Table 2). However, sepals were also affected in being wider than normal and sometimes fused at the base. Subsequent studies (Brewer *et al*. 2004) revealed that *PTL* acts as a boundary gene in the sepal whorl, dampening growth between sepals. When extra intersepal growth occurs in *ptl* loss-of-function mutants, petal initiation nearby is disrupted (Lampugnani *et al*. 2012). This was related to auxin dynamics through a second-site mutagenesis screen of *ptl* single mutants that uncovered the auxin efflux gene *AUXIN1* as a supporter of petal initiation (Lampugnani *et al*. 2013).

A modifier of the *ptl* mutant phenotype, *PETAL LOSS MODIFIER* (*PMD*), was uncovered in un-mutagenized Landsberg *erecta* background that resulted in additional petals rather than fewer, with these petals arising with disrupted orientation within the flower meristem (Griffith *et al*. 1999). This variant was unique in being dominant, and its molecular nature has not yet been uncovered.

### Flower meristem size mutants: *clv1*, and *clv3*

One class of phenotypic mutants had flowers with increased numbers of all organs, especially carpels. In previous studies, this phenotype was named *clavata* (*clv*) after the club-shaped fruit with swelling at the apex (McKelvie 1962). Allelism tests showed that 2 occurrences obtained here were allelic with the *clv1-1* mutant already described (Koornneef 1983). Two further occurrences were shown not to be allelic with either *clv1-1* (Clark *et al*. 1993) or *clv2-1* (Kayes and Clark 1998), and were named *clv3-1* and *clv3-2* (Clark *et al*. 1995) (Table 2). Mutants of these 3 genes, *CLV1*, *2*, and *3*, have closely similar single and multiple mutant phenotypes indicating that they share the same ultimate function (Table 1).

### Inflorescence meristem mutants: *tfl1*

One category of gene with a mutant phenotype with abnormal flowers was named *TERMINAL FLOWER1* (*TFL1*) (Shannon and Meeks-Wagner 1991; Alvarez *et al*. 1992). This arose 4 times in the original hunt at Caltech, and thrice more subsequently at Monash University (Table 2). The 7 alleles showed a spectrum of increasingly severe abnormalities, but all shared the consumption of the inflorescence meristem sooner or later by floral organs. This was accelerated at higher temperatures. It was proposed that TFL1 is required to maintain inflorescence meristem identity by preventing it from taking on floral meristem identity (Alvarez *et al*. 1992). We speculated that TFL1 may play a role in the evolution of determinate inflorescence shoots.

### Auxin response mutants: *pin1* and *pid*

A final category of flower development mutants mostly lacked flowers on the pin-like inflorescence shoot. However, occasional flowers did form that were characteristically abnormal in the number and morphology of all floral organs (Bennett *et al*. 1995) (Table 2). Two mutants were allelic with *pin formed1-1* (*pin1-1*) already-characterized (Okada *et al*. 1991). *pin1* mutants have pleiotropic defects in cotyledon number (reduced) and leaf number (decreased). One allele obtained in the screen, *pin1-5*, had a weaker phenotype and has been useful in revealing the consequences of partial loss of PIN1 function (e.g. Sohlberg *et al*. 2006). Also, 2 *pin1* mutant alleles were recently used to assay the ancient conserved transport function of PIN in nonvascular plants (Fisher *et al*. 2023).

At the same time, 5 other mutants sharing pin-like inflorescences contrarily had increased cotyledon numbers and increased numbers of leaves. A series of allelism tests revealed that a second locus was involved, and the gene was named *PINOID* (*PID*), meaning “pin-like” (Bennett *et al*. 1995). An allelic series of increasing severity was obtained, and double mutants with an *auxin resistant1* (*axr1*) mutant showed that PID, like PIN1, is associated with auxin function.

## Discussion

### Most mutants obtained were in flower specific genes

By focusing our screening on mutants that changed only floral properties, we were mostly able to avoid genes with wider and more general functions in plant growth and development, including house-keeping genes. For example, genes controlling cell structure and cell division were not revealed. These were more often obtained in screens of earlier developmental events, especially during embryogenesis when wild-type gene products derived maternally may be active for some time after fertilization. When eventually no activity remains, the disruption could lead to seed lethality (Meinke and Sussex 1979) or sometimes embryo patterning defects (Mayer *et al*. 1991).

It is of interest that all the mutants obtained here were recessive. This is in contrast with collections of maize morphological mutants (Richardson and Hake 2022). However, the maize collection includes many defective plants arising in crop conditions where dominant mutants would be immediately obvious. Also, in many cases, we now know that the dominant maize mutants often result from mis-expression of genes rather than their loss-of-function. As here, screens of EMS mutants in maize yield mainly recessive mutations, as expected from single base changes predominantly in transcribed regions (Neuffer and Sheridan 1980).

### Predominant recovery of transcription factor genes

In 1988, we had no knowledge of the molecular nature of flower development genes. Possible roles for hormones had been considered (Meyerowitz *et al*. 1989), but upon subsequent cloning, the majority of genes were found to encode transcription factors. In hindsight, this was perhaps unsurprising given precedents from the cloning of animal developmental genes, especially those with homeotic mutational changes. Differences between homeotic gene classes in animals (encoding homeodomain family members of the HOX subfamily) and plants (encoding mostly MADS family members) were revealed, including their genomic organization (clustered in animals, dispersed in plants), their expression patterns (sequential along a physical gradient in animals, coincidental in concentric overlapping fields in plants), and the multimeric structure of their functional products (mono- or di-meric HOX proteins in animals, hetero-tetrameric MADS proteins in plants). But a common function is the regulation of target genes, still being defined. For example, CRC expression is indeed a direct target of AG action (Ó’Maoiléidigh *et al*. 2013), as proposed in the initial screen.

Apart from the MADS family, founder members of plant-specific families such as the AP2 family (Jofuku *et al*. 1994) and the YABBY family (Bowman and Smyth 1999) (Fig. 2e) were revealed. Many members of these families control morphogenetic processes, suggesting that they have arisen from neofunctionalization following duplication of an early common ancestor with such a role. Other genes encoding trihelix proteins (Brewer *et al*. 2004) and bHLH proteins (Heisler *et al*. 2001) occurred too (Table 2), but members of these large families have widely divergent roles suggesting more recent neo-functionalization leading to a floral role. On the other hand, the plant-specific *LFY* gene usually occurs as the sole member of its family (Moyroud *et al*. 2010), indicating that newly duplicated copies are unlikely to coexist because of mal-adaptation.

As an illustration of how our screen has led to a wider understanding of plant development, CRC was a founding member of the YABBY family (Bowman and Smyth 1999) along with FILAMENTOUS FLOWER (FIL) (Sawa *et al*. 1999). In addition to its role in fine-tuning carpel development, CRC was also revealed to be required for nectary development and to function with AG in promoting floral meristem determinacy. The strategy of screening for second-site mutants was also exploited in *crc* mutants (Page and Grossniklaus 2002) and led to uncovering a role for YABBY family members in defining top-bottom polarity of lateral organs, including leaves and carpels, and to identifying other gene families involved in this process (Eshed *et al*. 1999; Siegfried *et al*. 1999). Later studies have revealed other roles of CRC in supporting leaf midrib development in grasses (Yamaguchi *et al*. 2004), and in conferring sexual dimorphism in flowers of melons and cucumbers (Zhang *et al*. 2022), for example.

### New signaling genes

One important plant-specific signaling mechanism was uncovered in the *clavata* mutants. These are now known to limit the spread of stem cell identity from the organizing center of flower meristems as well as shoot and inflorescence meristems (Schoof *et al*. 2000). In particular, a novel entry point to signaling was provided by CLV3 (Fletcher *et al*. 1999; Brand *et al*. 2000), a founding member of the CLE family of mobile peptide signaling molecules, now known to be a predominant mode of signaling between plant cells (Willoughby and Nimchuk 2021).

Another signaling process is controlled by TFL1, a phosphatidylethanolamine binding protein (Bradly *et al*. 1997) that represses the floral nature of the inflorescence meristem. It does this antagonistically with the florigen FLOWERING LOCUS T (FT) from the same family. The *ft* mutant phenotype is late flowering [*tfl1* is early, Shannon and Meeks-Wagner (1991)] but *ft* flowers themselves are normal, and so mutants of this gene, and many other occurrences of late flowering mutants (e.g. Rédei 1962; Koornneef *et al*. 1991), were not followed up here. We proposed that TFL1 plays a role in the evolutionary switching of the inflorescence meristems between determinate and indeterminate growth forms (Alvarez *et al.* 1992), now being borne out by observation, for example in the woodland strawberry *Fragaria vesca* (Lembinen *et al*. 2023).

Our focus on abnormal flowers meant that we included *pin1* and *pid* mutant plants in which monstrous flowers sometimes arose on otherwise naked inflorescences. We discovered the *PID* gene through genetically separating *pid* from *pin1* mutants. This ultimately led to the discovery that the PID kinase phosphorylates PIN1 (Michniewicz *et al*. 2007), and to a continuing expansion of knowledge of auxin signaling.

## Conclusion

Forward genetic screens have been instrumental in providing an initial spectrum of genes for follow up by cloning and molecular characterization. In Arabidopsis, chemical mutagenesis methods were joined by transposon tagging (Sundaresan *et al*. 1995), activation tagging (Weigel *et al*. 2000), antisense RNA knock down (Chuang and Meyerowitz 2000), and large-scale T-DNA insertional mutagenesis (Feldmann *et al*. 1989; Alonso *et al*. 2003) as a source of developmental genes. These could generate mutant phenotypes per se, or changes in reporter gene expression. Once the DNA sequence of candidate genes was known, large-scale libraries of such resources could be screened, in pools if needed, to obtain additional mutant versions of the gene of interest. One disadvantage of insertional mutagenesis was the predominance of loss-of-function mutant alleles, but this was remedied to some extent by screening of Targeted Induced Local Lesions in Genomes populations (McCallum *et al*. 2000). A recent revolution in obtaining knock outs of genes of known sequence has been provided by CRISPR-Cas9 methods (Zhang *et al*. 2019), although recovery of partial loss-of-function alleles is not yet straightforward. In general, genomic resources are now widespread and accessible, but our understanding of a gene's function still depends to a large extent on modifying its expression.

Developmental gene discovery has been very successful in Arabidopsis. Once cloned, the role of each gene and its paralogs has been followed up in Arabidopsis using expression analysis, multiple mutant studies, and reverse genetics approaches, especially in the large gene families characteristic of many transcription factors. Together with further regulatory and signaling genes uncovered by us and others, gene regulatory networks of flower development in Arabidopsis are now one of the most complete developmental pathways yet defined for flowering plants (Chen *et al*. 2018; Thomson and Wellmer 2019; Zúñiga-Mayo *et al*. 2019).

Also, findings have been extended to both orthologs and paralogs in other species, leading to the burgeoning field of evo-devo (Smyth 2018; Kramer 2019). One satisfying result of initially focusing on one model species, Arabidopsis, has been the finding that the role of core morphogenetic genes is often conserved across flowering plants. This early assumption has fortunately borne fruit. Even so, exceptions are to be treasured as helping build a more complete and balanced picture of exactly how the exceptional diversity of floral forms has evolved.
